# Effect of sirolimus on urinary bladder cancer T24 cell line

**DOI:** 10.1186/1756-9966-28-3

**Published:** 2009-01-07

**Authors:** Rosario Pinto-Leite, Pedro Botelho, Eufemia Ribeiro, Paula A Oliveira, Lucios Santos

**Affiliations:** 1 Genetic Service, Cytogenetic laboratory, Hospital center of Trás-os-Montes and Alto Douro, 5000-508 Vila Real, Portugal; 2 CECAV, Department of Veterinary Sciences, CECAV, University of Trás-os-Montes and Alto Douro, 5001-911, Vila Real, Portugal; 3 Department of Surgical Oncology, Portuguese Institute of Oncology, Porto, Portugal; 4 Health Faculty, Fernando Pessoa University, Porto, Portugal

## Abstract

**Background:**

Sirolimus is recently reported to have antitumour effects on a large variety of cancers. The present study was performed to investigate sirolimus's ability to inhibit growth in T24 bladder cancer cells.

**Methods:**

T24 bladder cancer cells were treated with various concentrations of sirolimus. MTT assay was used to evaluate the proliferation inhibitory effect on T24 cell line. The viability of T24 cell line was determined by Trypan blue exclusion analysis.

**Results:**

Sirolimus inhibits the growth of bladder carcinoma cells and decreases their viability. Significant correlations were found between cell proliferation and sirolimus concentration (r = 0.830; p < 0.01) as well as between cell viability and sirolimus concentration (r = -0.896; p < 0.01).

**Conclusion:**

Sirolimus has an anti-proliferation effect on the T24 bladder carcinoma cell line. The information from our results is useful for a better understanding sirolimus's anti-proliferative activity in the T24 bladder cancer cell line.

## Background

Bladder cancer is the second most common urologic malignancy and accounts for approximately 90% of cancers of the urinary tract. Is the fourth most incident cancer in male and ninth in females [1]. In industrialized countries, more than 90% of cases are originate in the urothelial epithelial cells (called urothelial cell carcinoma) [2]. At diagnosis, 75% are non-invasive bladder cancer. The invasive bladder cancers may spread outside the bladder and affect other organs. Bladder cancer's staging, treatment and prognosis depend on how deeply it has invaded urinary bladder [3].

Fortunately, about 80% of patients with non-muscle invasive disease can be successfully treated using the surgery. Historically, two-thirds of patients have tumour recurrence within 5 years. High-grade tumours have a significantly worse prognosis. Both high-grade T1 tumours and carcinoma *in situ *have the potential to progress and even metastasize [4]. Patients with invasive bladder cancer require a radical cystectomy. Controversy exists as to whether neoadjuvant or adjuvant chemotherapy improves survival in patients with invasive bladder cancer, despite a number of randomised controlled trials. So far there are no data to confirm what is the best combination of treatments (neoadjuvant chemotherapy, adjuvant with or without radiotherapy) to treat invasive bladder cancer [5]. The modest results with currently drugs, suggest the urgent need to identify new agents [6]. Sirolimus is a macrocyclic lactone that was first discovered as a product of the soil bacteria *Streptomyces hygroscopicus*. It was originally used as an immunosuppressant drug to help prevent rejection in organ transplantation, particularly in kidney transplant operations, but the authors of a number of recent reports have indicated that it may have other potential biological effects as an anti-cancer medicine [7,8]. Both the immunosuppressive and anti-cancer properties of sirolimus are due to the inhibition of the mammalian target of the sirolimus (mTOR) signalling pathway, which controls mRNA translation and induces angiogenesis and cell proliferation. Angiogenesis and a high proliferative index correspond to a poor prognosis for urothelial bladder cancer patients [9,10]. Sirolimus forms a complex with the immunophilin prolyl isomerase FK binding protein complex (FKBP-12) that binds with high affinity to mTOR [11,12]. This interaction inhibits mTOR kinase activity and subsequently decreases the phosphorylation of 4E binding protein-1 and the inhibition of the 40S ribosomal protein p70 S6 kinase [13-15]. Sirolimus's antineoplasic effects have been related to its capacity to inhibit the translation machinery involved in the regulation of G1- to S-phase transition in cell cycle [16,17]. Cell growth and proliferation in numerous cancer types are often regulated by the mammalian target of sirolimus (mTOR) pathway through p7056 kinase, ribosomal S6 protein, and eukaryotic initiation factor 4 E-binding protein 1 [18]. Recently there has been an enormous increase in our understanding of the molecular mechanisms underlying sirolimus's therapeutic anti-cancer properties. Alterations in the pathway regulating mTOR occur in many solid malignancies including bladder cancer. *In vitro *and *in vivo *models of bladder cancer have established the importance of the mTOR pathway in controlling cancer progression and metastasis [19]. The T24 cell line has been established from a highly malignant grade III human urinary bladder carcinoma [20]. This cell line can be easily grown *in vitro *and has been extensively used to evaluate the therapeutic effects of several anticancer drugs. Here, we describe the preliminary results of the study of the therapeutic effect of sirolimus against human T24 bladder cancer cell line *in vitro *using 3-(4,5-dimethylthiazol-2-yl)-2,5-diphenyltetrazolium bromide (MTT) assay for assessing cell proliferation and Trypan blue for assessing cell viability.

## Materials and methods

### Cell culture

Cell line T24 was provided by a German collection of microorganisms and cell cultures (DSMZ, Düsseldorf, Germany). Cells were grown as a monolayer in complete RPMI (RPMI-1640 medium supplemented with 10% fetal calf serum, 100 U/mL penicillin and l00 μg/mL streptomycin), in a humidified atmosphere with 7% CO_2_-93% air at 37°C. Under these conditions, the plating efficiency was 70–90% and the doubling time was 9–10 h. Single cell suspensions were obtained by trypsinization of monolayer cultures.

### Drugs

Sirolimus was purchased from Wyeth (Rapamune).

### Cell proliferation

The anti-proliferative capacity of the treatments was assessed by the MTT [21]. This is based on the reduction of MTT by mitochondrial dehydrogenase of intact cells to a purple formazan product. Using a Neubauer counting chamber cells were counted and 2 × 10^4 ^cells were seeded in 1 ml of medium in a 96-well culture plates and allowed to attach for 24 hours. Cells were treated with sirolimus (5 ng/mL, 10 ng/mL, 40 ng/mL, 60 ng/mL, 100 ng/mL, 150 ng/mL, 200 ng/mL, and 250 ng/mL) for 72 h, these doses were based on results published by other researchers [22,23]. Each of the concentrations above was regarded as one treated group while there was no sirolimus in the control group. After incubation, cell proliferation was evaluated by MTT assay according to the manufacturer's instructions. The MTT solution (20 μL, 5 mg/ml) was added to each well 3 h prior to the end of the 72 h chemical treatment exposure period. The media were removed at the end of the 72 h exposure period. The insoluble purple formazan crystals were dissolved in 100 μL DMSO/well and the absorbance was detected at 570 nm and 690 nm using a spectrophotometer (U 2000, Hitachi). The proliferation inhibitory rate percentage was calculated as follows: proliferation inhibitory rate (%)= 1-(A570-A690) of experimental wells/(A570-A690) of control wellsX100. Assays were performed in triplicate.

### Assay of cell viability

The viability of T24 cell line was determined by Trypan blue exclusion analysis. 0.2 ml of the cells suspension treated with sirolimus at various concentrations were transferred to test tubes with 0.5 ml of 0.4% Trypan blue solution and 0.3 ml of HBSS and mixed thoroughly. Allow to stand for 5 to 15 minutes. The percentage of viable cells was evaluated under the field microscope. Assays were performed in triplicate.

### Statistical analysis

All experiments were performed in triplicate and data were expressed as mean values ± SD. The Pearson product-moment correlation coefficient was used to evaluate the correlation (linear dependence) of cell proliferation, viability and sirolimus concentration. Data were analysed using SPSS 12 statistical software (SPSS Inc. USA) and statistical significance was set at p < 0.05.

## Results

### Cell proliferation

**Figure 1:**
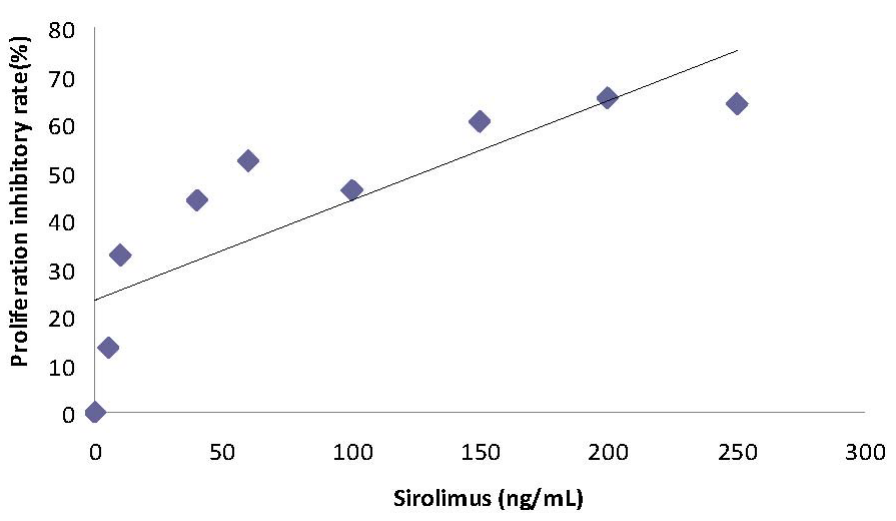
**Linear relationship between the proliferation inhibitory rate (%) and sirolimus concentration (y = 0.2074x + 23.299; r^2 ^= 0.6882)**.

The results of the MTT assay to detect sirolimus-induced anti-proliferative activity in T24 cell line are found in Table 1. T24 cancer cells were treated with various concentrations of sirolimus. As shown in Figure 1, sirolimus had growth inhibition effects on T24 cancer cells in a dose-dependent manner. Statistically, anti-proliferative activity was correlated with sirolimus concentration, the Pearson correlation of these two markers is r = 0.830 to p < 0.01.

**Table 1:** Effect of sirolimus in T24 cancer cell line.

Concentration	A570 nm	A690 nm	Mean ± SD
	0.525	0.201	
0 ng/mL	0.828	0.108	0.557 ± 0.207
	0.828	0.201	

	0.588	0.096	
5 ng/mL	0.639	0.078	0.481 ± 0.086
	0.72	0.33	

	0.528	0.054	
10 ng/mL	0.468	0.063	0.374 ± 0.117
	0.47	0.225	

	0.516	0.213	
40 ng/mL	0.489	0.087	0.310 ± 0.087
	0.477	0.25	

	0.78	0.489	
60 ng/mL	0.687	0.354	0.267 ± 0.080
	0.339	0.162	

	0.474	0.288	
100 ng/mL	0.573	0.246	0.301 ± 0.104
	0.657	0.267	

	0.501	0.276	
150 ng/mL	0.42	0.318	0.22 ± 0.115
	0.618	0.285	

	0.504	0.417	
200 ng/mL	0.294	0.255	0.193 ± 0.226
	0.576	0.123	

	0.345	0.264	
250 ng/mL	0.3	0.27	0.199 ± 0.249
	0.618	0.132	

### Cell viability

**Figure 2:**
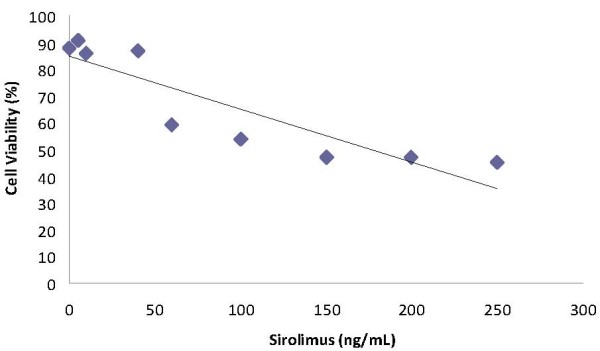
**Linear relationship between the cell viability rate (%) and sirolimus concentration (y = -0.1993x + 85.162; r^2 ^= 0.8023)**.

The results of cell viability after the incubation of the T24 cell line with sirolimus at different concentrations are displayed in Figure 2. It can be seen from the figure that there was a concentration-dependent decrease in cell viability for all concentrations tested. A significant correlation was found between cell viability and sirolimus concentration (r = -0.896, p < 0.01).

## Discussion

The findings of the present study revealed that sirolimus inhibits T24 bladder cancer cell proliferation and decrease the cell viability including in clinical dose of this mTOR inhibitor. These data may be relevant if we remember the action of the mTOR pathway. mTOR is a 290 kDa serine-threonine kinase that regulates both cell growth and cell cycle progression through its ability to integrate signals from nutrient and growth factor stimuli [24]. Tumour angiogenesis may depend on mTOR signalling. Hypoxia induces production of vascular endothelial cell growth factor (VEGF) by tumour and stromal cells which may be partly controlled by mTOR signaling and through PI3K-Akt-mTOR pathway [25]. Thus, the anti-tumour effects noted by inhibiting mTOR may be related to antiproliferative effects within tumour cells as well endothelial cells. Upstream effectors that signal through mTOR may up-regulate mTOR gene. Wu X et al (2004) showed that a specific inhibitor of PI3 kinase enzyme activity, Ly294002, potently suppressed the invasive properties of three highly invasive bladder tumour cell lines and 55% of primary tumours from patients with bladder cancer had markedly high levels of phosphorylated Akt [26]. Thus, the inhibition of mTOR may inhibit abnormal cell proliferation, tumour angiogenesis, and abnormal cell metabolism and potentially enhance the efficacy of other cancer treatments. The biological mechanisms responsible for anti-proliferative effect of sirolimus and the role of PI3K-Akt-mTOR pathway are under investigation [27]. Tanaka and Grossman (2003) demonstrate that PTEN can induce growth suppression and increase sensitivity to doxorubicin in bladder cancer cells and suggest that the PTEN gene and its pathways can be therapeutic targets for bladder cancer. To emphasize that, no results have been yet published on the activity of mTOR inhibitors against T24, or other, bladder cancer cell lines. Luan FL et al. (2002) showed that, sirolimus treatment alone, or with cyclosporine, prolonged the survival of mice inoculated with renal cancer cells or T24 human bladder cancer cells [28]. This is an indirect assumption of sirolimus effect against T24. The present study is the first address this issue. In the other hand, our team observed similar results, when we studied the effects of sirolimus in chemical induced urothelial cancers in ICR mice (data submitted). Sirolimus has been shown to inhibit the proliferation of various tumour cell lines including rhabdomyosarcoma, neuroblastoma, glioblastoma, small cell lung cancer, osteosarcoma, pancreatic cancer, breast cancer, prostate cancer, murine melanoma, leukaemia, and B-cell lymphoma [29-33].

Sirolimus enhances the anti-tumour effect of gemcitabine [34]. Now we intend to verify the efficacy of sirolimous mTOR inhibition, in other bladder cancer cell lines (5637, HT1376 and MC). Clinical results show that mTOR inhibitors are well tolerated and may induce prolonged stable disease and tumour regression in cancer patients [24]. Urgent research is needed to evaluate the real place of sirolimus or similar drugs in urothelial bladder cancer therapeutic.

## Conclusion

Sirolimus inhibits T24 bladder cancer cell proliferation and decrease cell viability including in clinical dose, therefore should be considered to be a promising agents against bladder cancer. However, more positive data will be necessary.

## Competing interests

The authors declare that they have no competing interests.

## Authors' contributions

PLR and BP carried out cell cultures, performed the statistical analysis and drafted the manuscript, RE participated in its design, OPA helped to draft the manuscript and revised the manuscript, SL supervised experimental work and revised the manuscript. All authors read and approved the final manuscript.
